# Supplementary material for: Cytosolic Hsp70 and co-chaperones constitute a novel system for tRNA import into the nucleus
Source: eLife. 2015 Apr 8;4:e04659. doi: 10.7554/eLife.04659 (PMC4432389; doi:10.7554/eLife.04659)
Supplement: Figure 3—source data 1. — Yeast cells are processed as described in the Figure 3 legend and the ‘Materials and methods’ section. Images from three independent sets of FISH experiments are subjected to quantification. Each folder named as Fig3_expX contains gray-scale tif images with 16 bit depth (acquired by MetaMorph) of a set of the experiments. A file name consists of the strain name (‘wt,’ ‘ssa1,’ for example) and culture conditions (‘YPD’ or ‘SD’) with the last capital letter representing the recording channel (‘D’ for DAPI staining or ‘R’ for RNA FISH). If the number of cells suitable for quantification in one image was under 30, those from two images were quantified. In such cases, two sets of images (‘ssa2_SD_a_R.tif’ and ‘ssa2_SD_b_R.tif’ for example) are included. Raw quantification data and their processing to NAIs are summarized Excel files. Summary of the total experiments are shown in the ‘SUMMARY’ sheet in the file named ‘Figure 3_data_summaryandexp1_DATA.xls.’ All the tif images have 16-bit depth. DOI: http://dx.doi.org/10.7554/eLife.04659.011 [file elife04659s002.zip › Figure 3 source data/Fig 3 material explanation.docx]

Yeast cells are processed as described in the Figure 3 legend and the Experimental Procedures section. Images from three independent sets of FISH experiments are subjected to quantification. Each folder named as Fig3_exp**X** contains gray-scale tif images with 16 bit depth (acquired by MetaMorph) of a set of the experiments. A file name consists of the strain name ("wt," "ssa1," for example) and culture conditions ("YPD" or "SD") with the last capital letter representing the recording channel ("D" for DAPI staining or "R" for RNA FISH). If the number of cells suitable for quantification in one image was under 30, those from two images were quantified. In such cases, two sets of images ("ssa2_SD_a_R.tif" and "ssa2_SD_b_R.tif" for example) are included. Raw quantification data and their processing to NAIs are summarized Excel files. Summary of the total experiments are shown in the "SUMMARY" sheet in the file named "Figure 3_data_summary&exp1_DATA.xls." All the tif images have 16-bit depth.
