# Supplementary material for: Cytosolic Hsp70 and co-chaperones constitute a novel system for tRNA import into the nucleus
Source: eLife. 2015 Apr 8;4:e04659. doi: 10.7554/eLife.04659 (PMC4432389; doi:10.7554/eLife.04659)
Supplement: Supplementary file 1. — Plasmids used for in vitro transcription in this study. DOI: http://dx.doi.org/10.7554/eLife.04659.022 [file elife04659s006.doc]

**Table S1. Plasmids used for *in vitro* transcription in this study**

Name Vector Gene of Interest

pTYE326 pUC119 *T7p::mature tRNA-Pro_UGG_* *without CCA*

pTYE341 pUC119 *T7p::mature tRNA-Phe_GAA_*

pTYE346 pUC119 *T7p::mature tRNA-Pro_UGG_*

pTYE349 pUC119 *T7p::mature tRNA-Trp_CCA_*

pTYE356 pUC119 *T7p::pre-tRNA-Pro_UGG_ with CCA*

pTYE467 pUC119 *T7p::(AUUUA)_5_**

pTYE468 pUC119 *T7p::(ACCCA)_5_**

pTYE516 pUC119 *T7p::tRNA-Pro_UGG_-G18A/U54C*

pTYE517 pUC119 *T7p::tRNA-Pro_UGG_-C69G*

pTYE554 pUC119 *T7p::tRNA-Pro_UGG_-C70G*

pTYE555 pUC119 *T7p::tRNA-Pro_UGG_-G68C*

pTYE556 pUC119 *T7p::tRNA-Pro_UGG_-C67G*

pTYE557 pUC119 *T7p::tRNA-Pro_UGG_-C69GC70G*

pTYE558 pUC119 *T7p::tRNA-Pro_UGG_-C69GC70G*

pTYE559 pUC119 *T7p::tRNA-Pro_UGG_-C69G/G3C*

pTYS560 pUC119 *T7p::tRNA-Pro_UGG_-G68C/C4G*

pTYE561 pUC119 *T7p::tRNA-Pro_UGG_-C67G/G5C*

pTYE562 pUC119 *T7p::tRNA-Pro_UGG_-C69GC70G/G2CG3C*

* These sequences originated from Henics *et al.* (1999).
