# Supplementary material for: Cytosolic Hsp70 and co-chaperones constitute a novel system for tRNA import into the nucleus
Source: eLife. 2015 Apr 8;4:e04659. doi: 10.7554/eLife.04659 (PMC4432389; doi:10.7554/eLife.04659)
Supplement: Supplementary file 2. — Yeast strains used in this study. DOI: http://dx.doi.org/10.7554/eLife.04659.023 [file elife04659s007.doc]

**Table S2 Yeast strains used in this study**

Name Genotype Reference

BY4741 *MAT***a** *his3∆1 leu2∆0 met15∆0 ura3∆0* Brachmann *et al.,* 1998

JJ160 *MAT***a** *GAL2 ura3-1 ade2-1 leu2-3,112 trp1-1* Johnson & Craig,

*his3-11,13 can1-100 met2-∆1 lys2-∆2 ∆ydj1::HIS3* 2000

PJ31-3A *MAT***a** *GAL2 ura3-1 ade2-1 leu2-3,112 trp1-1* Johnson & Craig,

*his3-11,13 can1-100 met2-∆1 lys2-∆2* 2000

Ssa1p-FLAG *MAT***a** *GAL2 ura3-1 ade2-1 leu2-3,112 trp1-1* This study

*his3-11,15 can1-100 SSA1-3×FLGA::CgHIS3*

ssa1∆ mtr10 *MAT***a** *GAL2 ura3-1 ade2-1 leu2-3,112 trp1-1* This study

*his3-11,15 can1-100 ssa1∆::TRP1*

*CgHIS3::GAL7p::MTR10*

Ssa2p-FLAG *MAT***a** *GAL2 ura3-1 ade2-1 leu2-3,112 trp1-1* This study

*his3-11,15 can1-100 SSA2-3×FLGA::CgHIS3*

ssa2∆ mtr10 *MAT***a** *GAL2 ura3-1 ade2-1 leu2-3,112 trp1-1* This study

*his3-11,15 can1-100 ssa2∆::TRP1*

*CgHIS3::GAL7p::MTR10*

SWY1353 *MAT***α** *GAL2 ura3-1 ade2-1 leu2-3,112 trp1-1* Ho *et al.*, 1997

*his3-11,15 can1-100 snl1∆::HIS*

TYSC512 *MAT***a** *GAL2 ura3-1 ade2-1 leu2-3,112 trp1-1* Takano *et al.*, 2005

*his3-11,15 can1-100 ∆los1::URA3 ∆msn5::KanMX4*

TYSC612 *MAT***a** *GAL2 ura3-1 ade2-1 leu2-3,112 trp1-1* Takano *et al.*, 2005

*his3-11,15 can1-100 CgHIS3::GAL7p::MTR10*

TYSC918 *MAT***a** *GAL2 ura3-1 ade2-1 leu2-3,112 trp1-1* This study

*his3-11,15 can1-100 ssa1∆::CgHIS3*

TYSC920 *MAT***a** *GAL2 ura3-1 ade2-1 leu2-3,112 trp1-1* This study

*his3-11,15 can1-100 ssa2∆::CgHIS3*

TYSC950 *MAT***α** *GAL2 ura3-1 ade2-1 leu2-3,112 trp1-1* This study

*his3-11,13 can1-100 met2-∆1 lys2-∆2 ∆sis1::LEU2/*

pYW65 [*TRP1 CEN SIS1*]

TYSC951 *MAT***α** *GAL2 ura3-1 ade2-1 leu2-3,112 trp1-1* This study

*his3-11,13 can1-100 met2-∆1 lys2-∆2 ∆sis1::LEU2/*

pYW65 [*TRP1 CEN sis1-121*]

TYSC981 *MAT***a** *GAL2 ura3-1 ade2-1 leu2-3,112 trp1-1* This study

*his3-11,15 can1-100 ssa3∆::CgHIS3*

(Table 2, continued)

TYSC983 *MAT***a** *GAL2 ura3-1 ade2-1 leu2-3,112 trp1-1* This study

*his3-11,15 can1-100 ssa4∆::CgHIS3*

TYSC1013 *MAT***a** *GAL2 ura3-1 ade2-1 leu2-3,112 trp1-1* This study

*his3-11,15 can1-100 ssa1∆::CgHIS3 ssa2∆::CgTRP1*

TYSC1046 *MAT***a** *GAL2 ura3-1 ade2-1 leu2-3,112 trp1-1* This study

*his3-11,15 can1-100 ssa1∆::CgHIS3 ssa2∆::CgTRP1*

*los1∆::URA3 msn5∆::kanMX4*

TYSC1059 *MAT***a** *GAL2 ura3-1 ade2-1 leu2-3,112 trp1-1* This study

*his3-11,15 can1-100 ssa1∆::CgHIS3 los1∆::URA3*

*msn5∆::kanMX4*

TYSC1060 *MAT***a** *GAL2 ura3-1 ade2-1 leu2-3,112 trp1-1* This study

*his3-11,15 can1-100 ssa2∆::CgTRP1 los1∆::URA3*

*msn5∆::kanMX4*

W303-1A *MAT***a** *GAL2 ura3-1 ade2-1 leu2-3,112 trp1-1* Thomas &

*his3-11,15 can1-100* Rothstein, 1989

W303-1B *MAT***α** *GAL2 ura3-1 ade2-1 leu2-3,112 trp1-1* Thomas &

*his3-11,15 can1-100* Rothstein, 1989

WY26 *MAT***α** *GAL2 ura3-1 ade2-1 leu2-3,112 trp1-1*  Yan & Craig,

*his3-11,13 can1-100 met2-∆1 lys2-∆2 ∆sis1::LEU2/* 1999

pYW17 [*URA3 CEN SIS1*]

5937 *MAT***a** *his3∆1 leu2∆0 met15∆0 ura3∆0* *zuo1∆::kanMX4* Open Biosystems
